# How do patients die in a rehabilitative unit dedicated to advanced respiratory diseases?

**DOI:** 10.1186/2049-6958-7-18

**Published:** 2012-07-20

**Authors:** Michele Vitacca, Laura Comini

**Affiliations:** 1 Pulmonary Rehabilitation Division, Salvatore Maugeri Foundation, IRCCS, Scientific Institution of Lumezzane, Via Giuseppe Mazzini, 129, 25066, Lumezzane, Brescia, Italy; 2 Healthcare Division, Salvatore Maugeri Foundation, IRCCS, Scientific Institution of Lumezzane, Via Giuseppe Mazzini, 129, 25066, Lumezzane, Brescia, Italy

**Keywords:** End of life, Mechanical ventilation, Palliative care

## Abstract

**Background:**

Evidences on how in-hospital COPD patients are cared in a Rehabilitative Respiratory Unit during the last time before death are lacking. This observational study was aimed at 1. analyzing the characteristics of respiratory patients who die in a Rehabilitative Unit dedicated to advanced care; 2. studying the available organizational support related to the dying process and quality of care in the last week of life.

**Methods:**

Medical records (MR) of patients suffering from respiratory disease admitted to a Rehabilitative Respiratory Unit during the last seven years (2005–2011) were collected retrospectively. Only MR of patients who died of respiratory complications were considered. This study describes clinical and demographic variables or information about drugs, procedures, health and unprofessional teams, intervention and interaction, habits and wishes in the last week of life.

**Results:**

110 patients out of 2,615 subjects (4.2%) died during the period of observation. 87 out of 110 patients fulfilled the inclusion criteria. They were aged, males, retired, severely compromised, with previous stays in an acute hospital and with a long stay in our unit. Most of them were married, lived in a small village and were cared at home by a caregiver. One third of patients came from Intensive Care Units. During the last week of life, hours spent under mechanical ventilation were extremely high both for patients under invasive (22.3 ± 3.1 hours) and non invasive ventilation (NIV) (17.5 ± 3.4 hours). The number of patients who maintained NIV was twice that of the intubated ones. Breathlessness and secretion encumbrance were the main symptoms. Secretion management was necessary in more than 50% of the cases. Communication between patient and doctor was good in the majority (67%) of the cases. Patient’s and family wishes, aimed at improving their relationships, were obtained in a high percentage (63%) of the cases. Doctors prescribed sedative in a half of patients and morphine only in 40% of the cases. Patients mainly died for acute respiratory failure (55%) or infective complications (34%), almost all under mechanical ventilation. Only a minority of them (28%) reported to have had a discussion about end-of-life care with their physician; palliative/end of life decisions were taken in 13% of the cases. Sustaining figures such as psychologist (17%) or clergy (13%) were marginally required.

**Conclusions:**

The current data have confirmed that, also in a Rehabilitative Respiratory setting, quality of end-of-life care and patient-physician communication need further improvement.

## Background

Millions of people die because of advanced respiratory diseases and in particular of chronic obstructive pulmonary disease (COPD) [1] each year, quite often after a prolonged functional decline accompanied by a lot of suffering [2,3].

COPD patient’s death often occurs because of a sudden and unexpected episode of exacerbation [4]. Indeed, 2/3 of respiratory deaths occur in institutions and most commonly in hospitals for acute patients [5]*.* The more frequent cause of death is acute respiratory failure, and the age, male gender, comorbidities, previous hospitalizations for chronic respiratory failure (CRF), and admission to a ward not appropriate to treat respiratory disease, are the most important predictors of mortality [6].

Significant differences in mortality may exist among hospitals for acute patients, ranging between 11 and 40%: a higher mortality occurs when the presence of doctors is limited, few patients are treated and cared by a specialist physician [7], there is a NIV necessity, an acidotic status arises late after admission, [8] and pneumonia develops [9].

Previous literature in respiratory patients has studied extensively their time before death [10,11] showing that they usually present dyspnoea, fatigue, weakness, low mood and pain, with a poor control of symptoms. They stay much time at home and are often admitted to hospital presenting sufficient awareness of death risk [12,13].

Nevertheless, it is well known that patients who died from COPD lacked surveillance and received inadequate services from primary and secondary care in the year before death [12,13] causing a very significant burden to caregivers [14] both for the economic and emotional aspects.

Data regarding end-of-life care in respiratory patients on home mechanical ventilation have been published in a previous paper by our group [15] and they showed that these patients were aware of their prognosis and disease severity since well informed by their doctors. Caregivers reported that the relief of respiratory symptoms was not easily achieved despite the use of drugs and sedatives [15].

Although clear statements and practical recommendations have been proposed to clinicians for providing palliative care to patients with advanced respiratory diseases and critical illnesses [16], this policy is not widely followed due to the absence of palliative care services both out and inside of the hospital.

Evidences on how respiratory and in particular COPD patients are cared during the last time before death spent in hospital, and in particular in a Rehabilitative hospital, are lacking.

The purposes of this retrospective and observational study were 1. to analyze the characteristics of respiratory patients who die in a Rehabilitative Unit dedicated to advanced care 2. to study the available organizational support related to the dying process and the quality of care in the last week of life.

Moreover, this study aims at identifying the elements that can improve care in the final stages of life in order to design care projects able to answer the real needs of these patients.

## Methods

### Characteristic of the rehabilitative hospital

Salvatore Maugeri Foundation, IRCCS, in Lumezzane (Brescia, Italy) is a private Institute involved into rehabilitation, needs of long term care weaning, and research for advanced chronic diseases. The public health system finances all costs incurred in health care through taxes. Patients have to pay no money for care.

### Patients

Medical records of patients suffering from respiratory disease admitted to the Rehabilitative Respiratory Unit during the last seven years of clinical activity (2005–2011) were retrospectively recorded for the current observational study. Only patients who died of respiratory complications were studied. Patients with neuromuscular, neuron-degenerative and oncological diseases or pulmonary fibrosis died of respiratory causes were excluded from the study.

The variables were recorded for all patients and in the last week before death the data were reviewed (Table 1).

**Table 1:** Variables recorded in all patients

**During the clinical activity (2005–2011)**
*age*
*gender*
*reported diagnosis (COPD, mixed, other)*
*diagnostic related group (DRG) complexity of care score*
*number of comorbidities*
*presence of gastrostomy*
*length of stay*
*location prior of the admission (hospital, home)*
*location of residence (town or village)*
*marital status (single, married, separated, divorced, widowed, not declared)*
*work status (disabled, domestic job, retired)*
*care at home (yes/no) as a consequence of illness/disability or old age*
**Data revised in the last week before death**
*number of diagnostic and therapeutic procedures*
*type of ventilation used (tracheostomy, endotracheal intubation, NIV)*
*time of ventilation (number of hours)*
*time of oxygen use (number of hours)*
*number of patients submitted to bronchoscopy*
*management of secretions (cough or manual assistance)*
*prevalence and scoring of symptoms (0 = no; 10 = maximum)*
*patient/doctor communication (easy, difficult)*
*drug consumption (morphine, benzodiazepine)*
*causes of death*
*patients discharged into a single room (yes/no)*
*family H 24 access (yes/no)*
*patients wishes fulfilled (yes/no)*
*involvement of a psychologist (yes/no)*
*involvement of a clergy (yes/no)*
*presence (yes/no), modalities (oral, written) of anticipated directives (yes/no)*
*do not resuscitate (DNR) order (yes/no)*

Informal consent to collect, treat and study all clinical data filled in the patient’s medical records had been obtained by each patient at admission in our hospital.

### Data analysis

A descriptive analysis of the different conditions derived by medical records was expressed as number, percentage or mean ± SD where indicated.

## Results and discussion

110 out of 2,615 patients (4.2%) died during a period of seven years. 87 out of 110 patients fulfilled the inclusion criteria. Table 2 shows the clinical and demographic data of the studied patients. They were aged, mainly males, retired, married, affected by COPD, with high hospital care complexity, coming from an hospital for acute patients, living in a small village, and cared at home by a caregiver. One third of these patients came from Intensive Care Units and they had already been tracheostomized at the time of admission due to prolonged weaning necessities.

**Table 2:** Clinical and demographic data of the studied patients

**Patients, n**	**87**
**Age, yrs (mean ± sd)**	75.5 ± 8.5
**Patients > 80 y,%**	25.2
**Female,%**	37
**Length of stay, days (mean ± sd)**	31 ± 41
**Marital status,%**	
*Married living with the partner*	56
*Single*	6
*Separated/divorced*	7
*Widowed*	26
*Not declared*	5
**Employment activity,%**	
*Retired*	81
*Full-time working at home*	9
*Disabled or unable to work*	10
**Cared at home due to illness/disability or old age,%**	
*No*	15
*Yes, by person in the household*	51
*Yes, by person not in the household*	34
**COPD,%**	45
**Mixed,%**	25
**Other,%**	30
**Comorbidities, n (mean ± sd)**	5.2 ± 0.8
**DRG care complexity score**^**§**^**(mean ± sd)**	2.5 ± 1.3
**Location prior admission,%**	
*Hospital*	83
*Home*	17
**Location of residence,%**	
*Town*	23
*Village*	77

legend – d, days; DRG, diagnostic related group; n, number; y, year.

^§^(0.5, minimum value: 4.4, maximum value).

Table 3 shows that several procedures have been proposed to patients in the last week of life.

**Table 3:** Procedures related to the patient in the last week before death

**Patients under invasive MV, %**	
*Tracheostomized*	33.2
*Endotracheal intubation*	14.8
**Time of invasive MV, hours (mean ± sd)**	22.3 ± 3.1
**Patients on NIV, %**	29.9
**Time of NIV, hours (mean ± sd)**	17.5 ± 3.4
**Patients on O**_**2**_**alone,%**	22.1
**Patients with gastrostomy,%**	10
**Secretion management, %**	
*Submitted to bronchoscopy*	14
*Manual Care*	17
*Mechanical Devices*	28
**Diagnostic Therapeutic Procedures at patient’s chart closure, n (mean ± sd)**	5.5 ± 0.8

legend - *MV*, mechanical ventilation; *NIV*, non invasive mechanical ventilation; O_2,_ oxygen.

The majority of them died on mechanical ventilation (MV) with prevalence of the invasive modality. The number of patients who maintained NIV was twice that of the intubated ones. The hours spent on mechanical ventilation were extremely high both for invasive and non invasive ventilation. Gastrostomy concerned a minority of patients. Secretion management was necessary in more than 50% of the cases by the use of bronchoscopy, manual care or mechanical devices.

Clinical data, drugs, end of life communication, involved personnel, and organizational choices during the last week of life are showed in Table 4. Breathlessness and secretion encumbrance were the main symptoms, doctors prescribed sedative in half of patients and morphine only in 40% of the cases. In the majority of cases patients died because of Acute Respiratory Failure (ARF), while infective complications were the second cause; palliative/end of life decisions were taken in 13% of patients. Communication between patient and doctor was good in the majority of cases although only in few an advanced decision plan was available. Sustaining figures as psychologist or clergy were marginal in the multidisciplinary care. Patient’s and family wishes, aimed at improving their relationships during in-hospital stay, were obtained in a high percentage of subjects; indeed a single room location was available only in 23% of cases.

**Table 4:** **Clinical data, drugs, end-of-life communication, involved personnel/organizational** choices **during last week of patient’s life**

Main symptoms* (mean ± sd)	
*Dyspnoea*	8.13 ± 1.13
*Abdominal pain*	2.08 ± 1.48
*Agitation*	5.47 ± 2.45
*Pain*	1.48 ± 1.55
*Secretions encumbrance*	6.96 ± 1.39
**Prescribed drugs, %**	
*Benzodiazepine*	54
*Morphine*	40
*H 24 oxygen*	92
**Causes of death, %**	
*Acute Respiratory Failure*	54.6
*Palliative decision*	8
*Pneumonia*	17
*Sepsis*	17
*Withdrawal from MV*	3.4
**Patient-doctor communication**	
*Easy, %*	67
*Difficult,%*	13
**Written Advanced Decision, %**	4.6
**Doctor/patient EOL speech, %**	28
**DNR order, %**	13
**Psychologist contact, %**	17
**Clergy contact, %**	13
**Single room availability, %**	23
**H 24 Family access, %**	85
**Patient’s wishes fulfilled, %**	63

legend - * rank of patient’s subjective sensation (0, no;10, maximum);

*DNR*, do not resuscitation; *EOL*, end of life; *MV*, mechanical ventilation.

This retrospective study presents lights and shadows regarding end-of-life care of patients admitted to a Rehabilitative Respiratory Unit dedicated to advanced respiratory diseases.

As expected, died patients were aged, males, retired, severely compromised, with previous experience in hospitals for acute patients, with a long stay in our unit, and the ARF was the more frequent cause of death. Interestingly, the majority of these patients were married, living in small villages, and cared at home by a caregiver.

Only few respiratory patients reported to have had a discussion about end-of-life care with their physician [14]. This was due to different reasons such as: too little time to discuss, unclear timing of discussion, psychological problems and necessities of repeated discussions.

Disease information, impact of symptoms, attitudes to receive help and unmet needs remained important causes of distress for respiratory patients [17].

Clinical condition, refuse, confusion, depression, and total pain may limit the interpretation of the patient’s wishes and these conditions might require a new organizational planning by care staff to approach patient and his family. In our experience testing patient's ability to communicate with doctors is a crucial issue: in order to overcome this important necessity, we have evaluated patient’s competence regarding the knowledge of his clinical situation through the involvement of his family. This latter allowed us to experience a better way to communicate patient’s care project especially when it had to be moved from ''cure'' to palliation.

Our data show that in the majority of cases, communication with patients was easy and difficult only in 13% of cases. The variability of care information witnesses the importance of an individual approach to patients with an apparently homogenous disease.

The alternative use of NIV has been suggested to patients and families who previously chose Endotracheal Intubation (EI) [11-19].

Excluding patients yet trachestomized at admission, 30% of our patients chose NIV, that was necessary for several hours per day. NIV was applied after careful discussion of its goals with explicit information on success and failure by experienced personnel and in an appropriate healthcare setting. Only few patients were submitted to EI.

In the Anglosaxon world, Do-Not-Resuscitate (DNR) order is the more frequent patients' preference (from 40 to 77%) [20] as the decision of not using mechanical ventilation (from 12 to 31%) [20]. COPD patients’ preferences (MV access, comfort assistance, DNR order) were similar to those of patients with cancer; however, COPD patients usually die in hospital more frequently under MV (70 vs 20%) [21], tube feed (39 vs 19%) [21], and after cardiopulmonary resuscitation (CPR) manouvres (25 vs 8%) [21].

In our study we reviewed the low percentage of patients with advanced care planning or DNR order, and we can confirm that, despite these patients were able to indicate their preferences regarding life sustaining treatments, burden and outcomes, their preferences were rarely discussed with their specialist physicians and more rarely were written for future sudden decisions [22].

Patients’ preferences regarding treatment choices may change over time. Our patients who experienced aggressive interventions (gastrostomy, tracheostomy or EI), more likely married and living in small villages, found these interventions acceptable for their life-extending.

The perceived family quality of dying and care have been studied, and they require some interventions and a more direct contact with the family members [23], as the family’s satisfaction often is poor after doctor’s speaking [24]. In our practice, information was provided on practical aspects (hospital visiting, accommodation, carers access) offering to the family free access to the patient and answering to patient’s requests as much as possible.

Patients with advanced COPD who die within 1 year have substantial comorbidities and symptoms [20]. Previous literature has confirmed that respiratory patients complain of heavy pain throughout the last period of their life while 2/3 have serious dyspnoea [15].

In the last three days before death, COPD patients suffer of severe dyspnoea, pain and confusion, and the breathless is the main symptom [25].

A previous study has implemented Integrated Care Pathways (ICPs) in order to set standards of care for symptom control in the last period of life [26]. Pain, agitation, and respiratory secretions have been controlled, so demonstrating that ICPs may provide a way to measure symptom control in dying patients [26].

Difficulties in palliating dyspnoea and secretion encumbrance were common in our patients. We revised the treatment of pain, agitation, and respiratory secretions by setting appropriate, although insufficient, prescription of sedative drugs and specific care.

Psychologists and clergy were rarely involved in our team due to under-estimation of psychological and religious needs, lack of personnel time or patient/family refuse. The religious tradition, if any, of both patient and family should be identified and pastoral support offered. In view of the patient's religion, any special needs, both around the time of dying and after death, should be identified. The current data have confirmed that quality of communication between patient and physician about end-of-life care needs further improvement [22].

## Conclusions

Our study presents some limitations, especially in relation to the sample size of the studied population and to the typology of the hospital site. However, these preliminary data suggest that the organization of health care and some aspects of the disease may play a role. However, the interpretation of these data should be made with caution, since the results refer only to data from the in-hospital medical records.

In order to understand the death rate (3.3%), clinical conditions and preferences of patients, it can be interesting to analyze the role of our reference center. It offers integrated rehabilitative programs to reduce dependencies in advanced patients with long history of chronic disease coming from nearby hospitals for acute patients. This peculiar “mission” might have influenced some of the shown/reported data, limiting a generalization of the results, but adding significant novelty to this field.

It is very difficult to establish criteria concerning the best place where to solve needs of respiratory patients in end of life stages. According to the local available resources, care plans to sustain patient’s necessities have to be established. Moreover, further studies are needed to individuate the organizational support necessary for respiratory clinicians to effectively deal with these issues. In particular, knowledge related to the process of dying and quality of care in the last days and hours of life should be improved. Finally, our study confirms that also in a Rehabilitative Unit patients affected with respiratory diseases do not always receive by clinicians the attention and care they should ideally have.

## Competing interests

The authors declare that they have no competing interests.

## References

[B1] Donner CF Nardini S The WHO Global Alliance Against Chronic Respiratory Diseases (GARD) launched in Bejing, March 2006 Multidiscip Resp Med 2006 1 2 8 10

[B2] Hansen-Flaschen J Chronic obstructive pulmonary disease: the last year of life Respir Care 2004 49 90 97 14733625

[B3] Lynn J Goldstein NE Advance care planning for fatal chronic illness: avoiding commonplace errors and unwarranted suffering Ann Intern Med 2003 138 812 818 12755553 10.7326/0003-4819-138-10-200305200-00009

[B4] Curtis JR Palliative and end-of-life care for patients with severe COPD Eur Respir J 2008 32 796 803 10.1183/09031936.00126107 17989116

[B5] Barclay S Arthur A Place of death: how much does it matter? The priority is to improve end-of-life care in all settings Br J Gen Pract 2008 58 229 231 10.3399/bjgp08X279724 18387225 PMC2277105

[B6] Faustini A Marino C D'Ippoliti D Forastiere F Belleudi V Perucci CA The impact on risk-factor analysis of different mortality outcomes in COPD patients Eur Respir J 2008 32 629 636 10.1183/09031936.00059807 18448492

[B7] Roberts CM Barnes S Lowe D Pearson MG Clinical Effectiveness Evaluation Unit, Royal College of Physicians; Audit Subcommittee of the British Thoracic Society. Evidence for a link between mortality in acute COPD and hospital type and resources Thorax 2003 58 947 949 10.1136/thorax.58.11.947 14586045 PMC1746518

[B8] Roberts CM Stone RA Buckingham RJ Pursey NA Lowe D National Chronic Obstructive Pulmonary Disease Resources and Outcomes Project implementation group. Acidosis, non-invasive ventilation and mortality in hospitalised COPD exacerbations Thorax 2011 66 43 48 10.1136/thx.2010.153114 21075776

[B9] Steer J Norman EM Afolabi OA Gibson GJ Bourke SC Dyspnoea severity and pneumonia as predictors of in-hospital mortality and early readmission in acute exacerbations of COPD Thorax 2012 67 117 121 10.1136/thoraxjnl-2011-200332 21896712

[B10] Goldberg AI Ethical basis of clinical decision at the end of life: long term home mechanical ventilation Multidiscip Resp Med 2007 2 4 101 103

[B11] Nava S Mechanical ventilation in the terminal respiratory patient Multidiscip Resp Med 2007 2 4 104 106

[B12] Elkington H White P Addington-Hall J Higgs R Edmonds P The healthcare needs of chronic obstructive pulmonary disease patients in the last year of life Palliat Med 2005 19 485 491 10.1191/0269216305pm1056oa 16218161

[B13] Elkington H White P Addington-Hall J Higgs R Pettinari C The last year of life of COPD: a qualitative study of symptoms and services Respir Med 2004 98 439 445 10.1016/j.rmed.2003.11.006 15139573

[B14] Vitacca M Escarrabill J Galavotti G Vianello A Prats E Scala R Peratoner A Guffanti E Maggi L Barbano L Balbi B Home mechanical ventilation patients: a retrospective survey to identify level of burden in real life Monaldi Arch Chest Dis 2007 67 142 147 18018753 10.4081/monaldi.2007.485

[B15] Vitacca M Grassi M Barbano L Galavotti G Sturani C Vianello A Zanotti E Ballerin L Potena A Scala R Peratoner A Ceriana P Di Buono L Clini E Ambrosino N Hill N Nava S Last 3 months of life in home-ventilated patients: the family perception Eur Respir J 2010 35 1 64 1071 10.1183/09031936.00172008 19717483

[B16] Lanken PN Terry PB Delisser HM Fahy BF Hansen-Flaschen J Heffner JE Levy M Mularski RA Osborne ML Prendergast TJ Rocker G Sibbald WJ Wilfond B Yankaskas JR ATS End-of-Life Care Task Force: An official American Thoracic Society clinical policy statement: palliative care for patients with respiratory diseases and critical illnesses Am J Respir Crit Care Med 2008 177 912 927 10.1164/rccm.200605-587ST 18390964

[B17] Jones I Kirby A Ormiston P Loomba Y Chan KK Rout J Nagle J Wardman L Hamilton S The needs of patients dying of chronic obstructive pulmonary disease in the community Fam Pract 2004 21 310 313 10.1093/fampra/cmh317 15128695

[B18] Curtis JR Engelberg RA Nielsen EL Au DH Patrick DL Patient-physician communication about end-of-life care for patients with severe COPD Eur Respir J 2004 24 200 205 10.1183/09031936.04.00010104 15332385

[B19] Curtis JR Cook DJ Sinuff T White DB Hill N Keenan SP Benditt JO Kacmarek R Kirchhoff KT Levy MM Society of Critical Care Medicine Palliative Noninvasive Positive VentilationTask Force: Non invasive positive pressure ventilation in critical and palliative care settings: understanding the goals of therapy Crit Care Med 2007 35 932 939 10.1097/01.CCM.0000256725.73993.74 17255876

[B20] Lynn J Ely EW Zhong Z McNiff KL Dawson NV Connors A Desbiens NA Claessens M McCarthy EP Living and dying with chronic obstructive pulmonary disease J Am Geriatr Soc 2000 48 5 Suppl S91 S100 10809462 10.1111/j.1532-5415.2000.tb03147.x

[B21] Claessens MT Lynn J Zhong Z Desbiens NA Phillips RS Wu AW Harrell FE Connors AF Dying with lung cancer or chronic obstructive pulmonary disease: insights from SUPPORT. Study to Understand Prognoses and Preferences for Outcomes and Risks of Treatments J Am Geriatr Soc 2000 48 5 Suppl S146 S153 10809468 10.1111/j.1532-5415.2000.tb03124.x

[B22] Janssen DJ Spruit MA Schols JM Wouters EF A call for high-quality advance care planning in outpatients with severe COPD or chronic heart failure Chest 2011 139 1081 1088 10.1378/chest.10-1753 20829337

[B23] Curtis JR Treece PD Nielsen EL Downey L Shannon SE Braungardt T Owens D Steinberg KP Engelberg RA Integrating palliative and critical care: evaluation of a quality-improvement intervention Am J Respir Crit Care Med 2008 178 269 275 10.1164/rccm.200802-272OC 18480429 PMC2542424

[B24] Tulsky JA Chesney MA Lo B How do medical residents discuss resuscitation with patients? J Gen Intern Med 1995 10 436 442 10.1007/BF02599915 7472700

[B25] Lynn J Teno JM Phillips RS Wu AW Desbiens N Harrold J Claessens MT Wenger N Kreling B Connors AF Perceptions by family members of the dying experience of older and seriously ill patients. SUPPORT Investigators. Study to Understand Prognoses and Preferences for Outcomes and Risks of Treatments Ann Intern Med 1997 126 97 106 9005760 10.7326/0003-4819-126-2-199701150-00001

[B26] Ellershaw J Smith C Overill S Walker SE Aldridge J Care of the dying: setting standards for symptom control in the last 48 hours of life J Pain Symptom Manage 2001 21 12 17 10.1016/S0885-3924(00)00240-2 11223310

